# Supplementary material for: Highly pathogenic avian influenza A (H5N1) in marine mammals and seabirds in Peru
Source: Nat Commun. 2023 Sep 7;14:5489. doi: 10.1038/s41467-023-41182-0 (PMC10484921; doi:10.1038/s41467-023-41182-0)
Supplement: Supplementary file 1 — Supplementary Information [file 41467_2023_41182_MOESM1_ESM.pdf]

Supplementary Table 1: Sampled animals and testing results, including genome coverage information by segment. NR: not recorded, M: male, F: female, U: unknown, A: adult, SA: sub-adult, J: juvenile, P: pup, N: newborn, n/a: not applicable.

ID	Region	Location	Collection date	Species	Sex	Age	Collection State	Sample Types Tested	Influenza-A Diagnostics			NGS % Coverage Length by Genomic Segment and Minimum Coverage Depth (x) Where Appropriate							
									PCR	Ct	Type	PB2	PB1	PA	HA	NP	NA	M	NS
1	Piura	Cangrejos - Paita	2022/11/22	*Delphinus delphis*	M	A	dead	Ocular conjunctival, rectal, spiracle swabs	+	30.24	H5N1	100 (54x)	100 (1x)	100 (1x)	100 (6x)	100 (76x)	100 (7x)	100 (9,555x)	100 (1,063x)
2	Piura	Cangrejos - Paita	2022/11/22	*Calidris alba*	U	J	euthanized	Cloacal, tracheal, lung, liver, brain swabs	+	14.97	H5N1	100 (66x)	100 (4x)	100 (46x)	100 (469x)	100 (1x)	100 (2x)	100 (414x)	100 (36x)
3	Piura	La Islilla - Paita	2022/11/23	*Pelecanus thagus*	M	A	dead	Lung, liver, tracheal, brain swabs	+	35.99	H5N1	100 (10x)	100 (91x)	100 (61x)	66	100 (1x)	100 (1x)	100 (2x)	100 (15x)
4	Piura	San Pedro de Vice	2022/11/24	*Pelecanus thagus*	M	A	dead	Lung, liver, spleen, tracheal, brain swabs	+	25.80	H5N1	100 (4x)	100 (3x)	100 (14x)	100 (57x)	100 (21x)	100 (211x)	100 (226x)	100 (407x)
5	Piura	San Pedro de Vice	2022/11/24	*Phalacrocorax bougainvillii*	F	A	euthanized	Lung, liver, spleen, tracheal, brain swabs	+	29.53	H5N1	100 (2x)	100 (1x)	100 (1x)	100 (1x)	100 (5x)	100 (49x)	100 (26x)	100 (51x)
6	Piura	San Pedro de Vice	2022/11/24	*Pelecanus thagus*	F	A	euthanized	Lung, liver, tracheal, brain swabs	+	16.18	H5N1	100 (325x)	100 (148x)	100 (331x)	100 (1073x)	100 (103x)	100 (403x)	100 (972x)	100 (486x)
7	Piura	San Pedro de Vice	2022/11/24	*Pelecanus thagus*	F	A	euthanized	Lung, liver, spleen, tracheal, brain swabs	+	30.64	H5N1	100 (17x)	100 (4x)	100 (192x)	84	100 (2x)	100 (30x)	100 (2x)	100 (131x)
8	Lima	Punta Roquitas - Miraflores	2023/01/18	*Otaria flavescens*	M	N	aborted	Lung, liver, spleen, thymus, stomach content swabs	+	20.03	H5N1	100 (27x)	100 (14x)	100 (12x)	100 (24x)	100 (17x)	100 (104x)	100 (348x)	100 (124x)
9	Lima	Chepeconde - Asia	2023/01/23	*Otaria flavescens*	H	SA	dead	Lung, liver, spleen swabs	+	36.11	n/a	0	0	0	0	0	17	0	0
10	Lima	Cayma - Asia	2013/01/24	*Otaria flavescens*	H	SA	alive	oral, nasal, rectal, oro-nasal swab	-	-	-	-	-	-	-	-	-	-	-

11	Lima	Punta Roquitas - Miraflores	2023/01/24	*Otaria flavescens*	H	A	euthanized	lung, spleen, liver swab	-	-	-	-	-	-	-	-	-	-	-
12	Lima	Playa Chica - San Bartolo	2023/01/25	*Otaria flavescens*	M	SA	alive	oral - rectal swab	+	36.47	H5N1	0	40	100 (11x)	99 (1x)	100 (2x)	100 (1x)	100 (6x)	100 (2x)
13	Lima	Miramar - Ancón	2023/01/26	*Otaria flavescens*	M	P	alive	oral-nasal-rectal swab	-	-	-	-	-	-	-	-	-	-	-
14	Lima	Conchán - Lurin	2023/01/31	*Otaria flavescens*	F	A	alive	feces	-	-	-	-	-	-	-	-	-	-	-
15	Lima	Playa Blanca - Asia	2023/02/02	*Spheniscus humboldti*	U	J	alive	oral, cloacal swab	-	-	-	-	-	-	-	-	-	-	-
16	Lima	Embajadores - Santa Maria del Mar	2023/01/28	*Otaria flavescens*	F	A	alive	oral, nasal, rectal swab	-	-	-	-	-	-	-	-	-	-	-
17	Lima	Sarapampa - Asia	2023/01/28	*Otaria flavescens*	F	A	alive	oral, nasal, rectal swab	-	-	-	-	-	-	-	-	-	-	-
18	Lima	Sarapampa - Asia	2023/01/28	*Otaria flavescens*	M	J	alive	oral, nasal, rectal swab	-	-	-	-	-	-	-	-	-	-	-
19	Lima	Barrancadero - Asia	2023/02/08	*Otaria flavescens*	F	NR	alive	nasal swab	-	-	-	-	-	-	-	-	-	-	-
20-24	Arequipa	Loberas - Caravelí	2023/02/07	*Otaria flavescens*	U	2P, 1J, 2A	2 dead 3 alive	pool of nasal swabs	+	29.48	H5N1	100 (47x)	100 (20x)	100 (171x)	100 (6x)	100 (1x)	100 (28x)	100 (235x)	100 (20x)
25	Tacna	Los Areanales - Tacna	2023/03/12	*Otaria flavescens*	M	J	alive	rectal swab	-	-	-	-	-	-	-	-	-	-	-
26	Tacna	El Chasqui - Yarada Los Palos	2023/03/12	*Larus belcheri*	U	J	dead	tracheal swab	-	-	-	-	-	-	-	-	-	-	-
27	Arequipa	Cerrillos - Camana	2023/03/06	*Phalacrocorax bougainvillii*	U	A	alive	oro-faringeal, cloacal swab	-	-	-	-	-	-	-	-	-	-	-
28	Arequipa	Catas -Islay	2023/03/06	*Otaria flavescens*	M	A	dead	nasal, oro-faringeal, rectal swab	+	34.72	H5N1	100 (5x)	100 (5x)	100 (6x)	27	33	32	70	54

Supplementary Table 2: SNP and mutational analysis of Peruvian HPAI a/H5N1 viruses. More than 70 variable sites were identified relative to the original A/H5N1 goose/Guangdong reference from 1996 and the A/Vietnam/1203/2004 reference used to annotate amino acid positions in the CDC inventory[23]. However, many of these sites are also present in more recent R6 strains isolated from birds (chicken/wyoming/2022) and mammals (skunk/washington/22-019274-001/2022) in 2022 (shaded in gray and Table 3). Mutations that have been previously reported as associated with specific pathogenic phenotypes of interest are shown with an asterisk (*). Mutations of interest in this study, defined as those that also differ from the original R6 reassortants that circulated in 2022 are shown in black. Of these, 7 are of particular interest as they appear more than once and are concentrated in mammals. n/d: no data; n/a not applicable.

Segment	Mutation	References		Sample ID and species											Associated phenotypes	References (CDC plus PMIDs)
		1996 Guangdong / 2004 Vietnam	2022 R6 bird / mammal	2	3	4	5	6	7	1	8	12	20-24	28		
				sandpiper	pelican	pelican	cormorant	pelican	pelican	dolphin	sea lion	sea lion	sea lion POOL	sea lion		
PB2	T215M	T	T	T	T	T	T	T	T	T	M	n/d	T	T	Only present in 1 sea lion	n/a
	I463V	I	V	V	V	V	V	V	V	V	V	n/d	V	V	Present in all genomes from Peru, plus Chile & Ecuador & 3 Colombia & 2 USA	n/a
	L464M	L	M	M	M	M	M	M	M	M	M	n/d	M	M	Present in all genomes from Peru, plus Chile & Ecuador & 3 Colombia & 2 USA & 1 Eurasia	n/a
	V478I	V	I	I	I	I	I	I	I	I	I	n/d	I	I	Present in all genomes from Peru, plus Chile & Ecuador & Venezuela & Colombia & 5 USA	n/a
	Q591K*	Q	Q	Q	Q	Q	Q	Q	Q	Q	Q	Q	Q	K	Only present in 1 sea lion and Chile human. Enhanced replication efficiency and increasedvirulence in mice	CDC, 31428925, 20700447, 26082035
	I616V	I	V/I	V	V	V	V	V	V	V	V	n/d	V	V	Present in all genomes from Peru, plus Chile & Ecuador & 3 Colombia & 1 USA	n/a
	E627K*	E/K	E	E	E	E	E	E	E	E	E	n/d	E	E	Present only in 2 mammals from Netherlands & 1 from USA. Altered polymerase activity, increased virulence	CDC, 19393699, 16533883, 19264775
	D701N*	D	D	D	n/d	D	D	D	D	D	D	n/d	N	N	Only present in 2 sea lions, 1 USA seal and Chile human. Mammalian adaptation	CDC, 19119420, 19264775
	K702R*	K	K	K	K	K	K	K	K	K	K	K	K	K	Not present in any genome. Mutation involved in host specificity and adaptation	28900145
	N715T	N	N	N	N	N	N	N	N	N	T	n/d	N	N	Only present in 1 sea lion	n/a
PB1	T59S	T	S	S	S	S	S	S	S	S	S	n/d	S	S	Present in all genomes from Peru, plus Chile & Ecuador & Colombia & 6 USA	n/a
	E264D	E	D	D	D	D	D	D	D	D	D	n/d	D	D	Present in all genomes from Peru, plus Chile &	n/a

														Ecuador & 3 Colombia & 2 USA	
	L378M	L	L	M	M	M	M	M	M	M	M	n/a	M	M	Present in all genomes from Peru & Chile	n/a
	G399D	G	D/G	D	D	D	D	D	D	D	D	n/d	D	D	Present in all genomes from Peru, plus Chile & 1 USA	n/a
	K429R	K	R	R	R	R	R	R	R	R	R	n/d	R	R	Present in all genomes from Peru, plus Chile & Ecuador & 3 Colombia & 2 USA	n/a
	S515A	S	S	S	S	S	S	S	S	S	S	A	S	A	Present in genomes of 2 sea lions & 5 Chile	n/a
PB1-F2	T7I	T	I	I	I	I	I	I	I	I	I	n/d	I	I	Present in all genomes from Peru, plus Chile & Ecuador & 3 Colombia & 6 USA	n/a
	S12L	S	L	L	L	L	L	L	L	L	L	n/d	L	L	Present in all genomes from Peru, plus Chile & Ecuador & 3 Colombia & 6 USA	n/a
	N17S	N	S	S	S	S	S	S	S	S	S	n/d	S	S	Present in all genomes from Peru, plus Chile & Ecuador & 3 Colombia & 2 USA	n/a
	R21K	R	K	K	K	K	K	K	K	K	K	n/d	K	K	Present in all genomes from Peru, plus Chile & Ecuador & Colombia & 6 USA & 2 Eurasia	n/a
	Y42C	Y	C	C	C	C	C	C	C	C	C	n/d	C	C	Present in all genomes from Peru, plus Chile & Ecuador & Colombia & 6 USA & 2 Eurasia	n/a
	S47N	S	N	N	N	N	N	N	N	N	N	n/d	N	N	Present in all genomes from Peru, plus Chile & Ecuador & Colombia & Venezuela & 7 USA & Eurasia	n/a
	R48Q	R/P	Q	Q	Q	Q	Q	Q	Q	Q	Q	n/d	Q	Q	Present in all genomes from Peru, plus Chile & Ecuador & Colombia & 6 USA & 3 Eurasia	n/a
	Q54R	Q	R	R	R	R	R	R	R	R	R	n/d	R	R	Present in all genomes from Peru, plus Chile & Ecuador & Colombia & 6 USA & 1 Eurasia	n/a
	I55T	I	T	T	T	T	T	T	T	T	T	n/d	T	T	Present in all genomes from Peru, plus Chile & Ecuador & Colombia & 6 USA	n/a
	W58L	W	L	L	L	L	L	L	L	L	L	n/d	L	L	Present in all genomes from Peru, plus Chile & Ecuador & Colombia & 6 USA	n/a
	N66S*	N	S	S	S	S	S	S	n/d	S	S	n/d	S	S	Increased virulence and replicacion efficiency	CDC, 31428925, 17922571, 21852950
	G70E	G	E/G	E	E	E	E	E	E	E	E	n/d	E	E	Present in all genomes from Peru, plus Chile & Ecuador & Colombia & 5 USA	n/a

PA	R57Q	R	R	R	R	R	R	R	R	R	R	Q	Q	Q	Present in genomes of 3 sea lions, plus 1 from Peru & Chile & Venezuela	n/a
	T61M*	T/I	M	M	M	M	M	M	M	M	M	M	M	M	Altered endonuclease activity	35127568, 27886255
	T85V	T	A	A	A	A	A	A	A	A	A	V	T	A	Mutation V present only in 1 sea lion	n/a
	M86I	M	M	M	M	M	M	M	M	M	I	M	M	I	Only present in 2 sea lions and Chile human	n/a
	M441V	M	V	V	V	V	V	V	n/d	V	V	V	V	V	Present in all genomes from Peru, plus Chile & Ecuador & Colombia & Venezuela & 8 USA	n/a
	T608S	T	S	S	S	S	S	S	S	S	S	S	S	S	Present in all genomes from Peru, plus Chile & Ecuador & Colombia & Venezuela & 8 USA	n/a
HA	D94S*	D	S	S	n/d	S	S	S	S	S	S	S	S	n/d	Increased virus binding to α2-6	CDC, 31428925, 19020946
	S123P*	S	P	P	n/d	P	P	P	P	P	P	P	P	n/d	Increased virus binding to α2-6	CDC, 31428925, 17108965
	S133A*	S	A	A	n/d	A	A	A	A	A	A	A	A	n/d	Increased virus binding to α2-6	CDC, 31428925, 17690300
	S155D*	S	D	D	n/d	D	D	D	n/d	D	D	D	D	n/d	Increased virus binding to α2-6	20844051, 19918095
	D183N	D	N	N	N	N	N	N	n/d	N	N	N	N	n/d	Increased virus binding to α2-6	CDC, 31428925, 22056389
	V210A*	V	A	A	n/d	A	A	A	n/d	A	A	A	A	n/d	Increased virus binding to α2-6	CDC, 31428925, 21637809
	K218Q*	K	Q	Q	n/d	Q	Q	Q	n/d	Q	Q	Q	Q	n/d	Increased virus binding to α2-3 & α2-6	31428925, 27869615
	S223R*	S	R	R	n/d	R	R	R	n/d	R	R	R	R	n/d	Increased virus binding to α2-3 & α2-6	31428925, 27869615
	Q322L*	Q	L	L	L	L	L	L	n/d	L	L	L	L	n/d	Cleavage Site	11148006
	R325K*	R	K	K	K	K	K	K	n/d	K	K	K	K	n/d	Cleavage Site	11148006
	H355R	H	H	H	H	H	H	H	H	H	H	R	R	n/d	Only present in 2 sea lions	n/a
	A496S	A	A	A	A	A	A	A	A	A	S	A	A	A	Only present in 1 sea lion	n/a
NP	M222L	M	M	M	M	M	M	M	M	M	L	M	M	n/d	Present in 1 sea lion	n/a
	F230L	F	L/F	L	L	L	L	L	L	L	L	L	L	L	Present in all genomes from Peru, plus Chile & 1 Colombia & 1 USA	n/a
	Y289F	Y	Y	Y	H	Y	Y	Y	Y	Y	Y	F	Y/F	n/d	Mutation F present in 2 sea lions and mutation H present in 1 pelican	n/a
	A428T	A	A	A	A	A	A	A	A	A	T	A	A	n/a	Only present in 1 sea lion	n/a
	S450N*	S	N	N	N	N	N	N	N	N	N	N	N	n/a	Eurasia has mutation S and North America has mutation N according to reference	23017273
	R452K	R	R	R	R	R	R	R	R	R	K	R	R	n/a	Only present in 1 sea lion & 1 from Eurasia	n/a
NA	I8T	I	T	T	T	T	T	T	T	T	T	T	T	n/a	Present in all genomes from Peru, plus Chile & Ecuador & Colombia & Venezuela & USA	n/a
	V62I	V	V	V	V	V	V	V	V	V	I	V	V	n/a	Only present in 1 sea lion	n/a
	A81I	A	T	T	T	T	I	I	T	T	T	T	T	n/a	Only present in 1 cormorant and 1 pelican	n/a

	L269M	L	M/L	M	M	M	M	M	M	M	M	M	M	n/a	Present in all genomes from Peru & Chile & 1 Colombia & 1 USA	n/a
	T289M	T	M	M	M	M	M	M	M	M	M	M	M	M	Present in all genomes from Peru, plus Chile & 5 Colombia & Venezuela & USA & Eurasia	n/a
	S339P	S	S	P	P	P	P	P	P	P	P	P	P	P	Present in most genomes from Peru & Chile	n/a
M1	N85S*	N	S/N	S	S	S	S	S	S	S	S	S	S	S	Present in all genomes from Peru, plus Chile & Colombia & USA. Mutation found in equine influenza	24224823
	N87T	N	N	T	T	T	T	T	T	T	T	T	T	T	Present in all genomes from Peru & Chile	n/a
	K101R*	K	R	R	R	R	R	R	R	R	R	R	R	R	Polymorphic region between human/avian strains, R is preffered in Human HPAIs	26537686
	A200V	A	V	V	V	V	V	V	V	V	V	V	V	n/d	Present in all genomes from Peru, plus Chile & Ecuador & Colombia & Venezuela & USA	n/a
M2	R61G	R	G	G	G	G	G	G	G	G	G	G	G	G	Present in all genomes from Peru, plus Chile & Ecuador & Colombia & Venezuela & USA	n/a
NS1	D26K	D/E	E	E	E	E	E	E	E	E	E	K	E	E	Only present in 1 sea lion	n/a
	A42S*	A/S	S	S	S	S	S	S	S	S	S	S	S	S	Increased virulence and pathogenicity in mammals	CDC, 31428925, 18032512
	E60V	E/A	A	A	A	A	A	A	A	A	A	V	A	A	Present in only 1 sea lion	n/a
	I81T	I	I	I	T	I	I	I	I	I	I	I	I	n/d	Present in 1 pelican & 1 Eurasia	n/a
	Y103F*	Y/F	F	F	F	F	F	F	F	F	F	F	F	n/d	Present in all genomes. Increased virulence in mice	CDC, 19052083, 21593152
	M116S	M/C	S/C	S	S	S	S	S	S	S	S	S	S	n/d	Present in all genomes from Peru, plus Chile & Ecuador & 1 Colombia & 1 USA	n/a
	I129M	I	I	I	I	I	I	M	I	I	I	I	I	n/d	Present in only 1 pelican	n/a
	D139N	D	N	N	N	N	N	N	N	N	N	N	N	n/d	Present in all genomes from Peru, plus Chile & Ecuador & 3 Colombia	n/a
	I205N*	I/N	S	N	S	S	S	S	S	S	S	S	N	S	Mutation N present in only 1 pelican & 1 sea lion. Mutation S present in all other genomes. Mutation S Decreases host antiviral response in ferrets	31428925, 20862325
	A223E	A	E/A	E	E	E	E	E	E	E	E	E	E	E	Present in all genomes from Peru, plus Chile & 3 Colombia & 1 USA	n/a

Supplementary Table 3: Primers used to generate sequences in this study

ID	SEQUENCE (5'-3')	AUTHOR	REFERENCE
CDC-InfA Fw1	CAAGACCAATCYTGTCACCTCTGAC	CDC	https://doi.org/10.3201/eid2707.210462
CDC-InfA Fw2	CAAGACCAATYCTGTCACCTYTGAC		
CDC-InfA Probe	TGCAGTCCTCGCTCACTGGGCACG		
CDC-InfA Rv1	GCATTYTGGACAAAVCGTCTACG		
CDC-InfA Rv2	GCATTTTGGATAAAGCGTCTACG		
Tuni-12	ACGCGTGATCAGCAAAAGCAGG	Zhou et al.	https://doi.org/10.1128/jvi.01109-09
Tuni-13	ACGCGTGATCAGTAGAAACAAGG		
	AGCRAAAGCAGGTCAATTATATTCA	Hurtado et al.	https://doi.org/10.1371/journal.pone.0145627
PB2-234R	AGTAGAAACAAGGTCGTTTTTAAACTA		
PB1-1	AGCRAAAGCAGGCAAACCATTTGAATG		
PB1-234R	AGTAGAAACAAGGCATTTTTTCATGAA		
PA-1	AGCRAAAGCAGGTACTGATYCGAAATG		
PA-2233R	AGTAGAAACAAGGTACTTTTTTGGACA		
Bm-HA-1	TATTCGTCTCAGGGAGCAAAAGCAGGGG	Hoffman et al.	https://doi.org/10.1007/s007050170002
Bm-NS-890R	ATATCGTCTCGTATTAGTAGAAACAAGGGTGTTTT		
Ba-NA-1	TATTGGTCTCAGGGAGCAAAAGCAGGAGT		
Ba-NA-1413R	ATATGGTCTCGTATTAGTAGAAACAAGGAGTTTTTT		

Supplementary Table 4: GenBank accession numbers for all sequences generated in this study. n/a = not applicable

	Genbank Accession Codes							
Sequence name	Seg 1	Seg 2	Seg 3	Seg 4	Seg 5	Seg 6	Seg 7	Seg 8
A/sanderling/Peru/PIU-SER005/2022	OQ550463	OQ550464	OQ550465	OQ550466	OQ550467	OQ550468	OQ550469	OQ550470
A/pelican/Peru/PIU-SER013/2022	OQ925705	OQ925706	OQ925707	OQ925704	OQ550419	OQ550420	OQ550421	OQ550422
A/pelican/Peru/PIU-SER019/2022	OQ550447	OQ550448	OQ550449	OQ550450	OQ550451	OQ550452	OQ550453	OQ550454
A/guanay cormorant/Peru/PIU-SER024/2022	OQ550423	OQ550424	OQ550425	OQ550426	OQ550427	OQ550428	OQ550429	OQ550430
A/pelican/Peru/PIU-SER028/2022	OQ550455	OQ550456	OQ550457	OQ550458	OQ550459	OQ550460	OQ550461	OQ550462
A/pelican/Peru/PIU-SER016/2022	OQ550431	OQ550432	OQ550433	OQ550434	OQ550435	OQ550436	OQ550437	OQ550438
A/common dolphin/Peru/PIU-SER002/2022	OQ550439	OQ550440	OQ550441	OQ550442	OQ550443	OQ550444	OQ550445	OQ550446
A/south american sea lion/Peru/LIM-SER036/2023	OQ550471	OQ550472	OQ550473	OQ550474	OQ550475	OQ550476	OQ550477	OQ550478
A/south american sea lion/Peru/LIM-SER00B/2023	n/a	OQ925716	n/a	OQ925717	OQ925718	OQ925719	OQ925720	OQ925721
A/south american sea lion/Peru/AQP-SER00R/2023	OQ925708	OQ925709	OQ925710	OQ925711	OQ925712	OQ925713	OQ925714	OQ925715
A/south american sea lion/Peru/AQP-SER00K/2023	OQ925722	OQ925723	OQ925724	OQ925725	OQ925726	OQ925727	OQ925728	OQ925729
