# Supplementary material for: Highly pathogenic avian influenza A (H5N1) in marine mammals and seabirds in Peru
Source: Nat Commun. 2023 Sep 7;14:5489. doi: 10.1038/s41467-023-41182-0 (PMC10484921; doi:10.1038/s41467-023-41182-0)
Supplement: Supplementary file 3 — Reporting Summary [file 41467_2023_41182_MOESM3_ESM.pdf]

nature portfolio

Corresponding author(s): Mariana Leguia

Last updated by author(s): Aug 15, 2023

Reporting Summary

Nature Portfolio wishes to improve the reproducibility of the work that we publish. This form provides structure for consistency and transparency in reporting. For further information on Nature Portfolio policies, see our Editorial Policies and the Editorial Policy Checklist.

Statistics

For all statistical analyses, confirm that the following items are present in the figure legend, table legend, main text, or Methods section.

n/a	Confirmed	
☐	☒	The exact sample size (*n*) for each experimental group/condition, given as a discrete number and unit of measurement
☒	☐	A statement on whether measurements were taken from distinct samples or whether the same sample was measured repeatedly
☒	☐	The statistical test(s) used AND whether they are one- or two-sided<br>*Only common tests should be described solely by name; describe more complex techniques in the Methods section.*
☒	☐	A description of all covariates tested
☒	☐	A description of any assumptions or corrections, such as tests of normality and adjustment for multiple comparisons
☒	☐	A full description of the statistical parameters including central tendency (e.g. means) or other basic estimates (e.g. regression coefficient) AND variation (e.g. standard deviation) or associated estimates of uncertainty (e.g. confidence intervals)
☒	☐	For null hypothesis testing, the test statistic (e.g. *F*, *t*, *r*) with confidence intervals, effect sizes, degrees of freedom and *P* value noted<br>*Give P values as exact values whenever suitable.*
☐	☒	For Bayesian analysis, information on the choice of priors and Markov chain Monte Carlo settings
☒	☐	For hierarchical and complex designs, identification of the appropriate level for tests and full reporting of outcomes
☒	☐	Estimates of effect sizes (e.g. Cohen's *d*, Pearson's *r*), indicating how they were calculated

Our web collection on statistics for biologists contains articles on many of the points above.

Software and code

Policy information about availability of computer code

Data collection	Complete descriptions are provided in main manuscript and in the supplementary materials. We gratefully acknowledge the authors and both originating and submitting laboratories of the sequences from GISAID's EpiFlu™ Database on which this research is based. The GISAID server was unable to download a large table of 4000+ bird viruses used in our analyses, but tables for all South American and mammalian viruses used are provided in the supplemental materials. We also provide GenBank accession numbers with hyperlinks for all the sequences generated as part of this study in Supplementary Table 4. In addition, XMLs, MCC and ML trees, and GISAID acknowledgement tables are also available in a GitHub repository (https://github.com/mostmarmot/Peru_AIV/).
Data analysis	Software used for data analysis has been specified in various sections of the materials and methods, with version used and references. For NGS data analysis we used a suite of bioinformatic tools, including BBDuk and SPAdes, that are contained within the Geneious Prime 2023.0.4 package. In addition, XMLs, MCC and ML trees, and GISAID acknowledgement tables are available in a GitHub repository (https://github.com/mostmarmot/Peru_AIV/).

For manuscripts utilizing custom algorithms or software that are central to the research but not yet described in published literature, software must be made available to editors and reviewers. We strongly encourage code deposition in a community repository (e.g. GitHub). See the Nature Portfolio guidelines for submitting code & software for further information.

Data

Policy information about availability of data

All manuscripts must include a data availability statement. This statement should provide the following information, where applicable:

- Accession codes, unique identifiers, or web links for publicly available datasets
- A description of any restrictions on data availability
- For clinical datasets or third party data, please ensure that the statement adheres to our policy

We gratefully acknowledge the authors and both originating and submitting laboratories of the sequences from GISAID's EpiFlu™ Database on which this research is based. The GISAID server was unable to download a large table of 4000+ bird viruses used in our analyses, but tables for all South American and mammalian viruses used are provided in the supplemental materials. We also provide GenBank accession numbers with hyperlinks for all the sequences generated as part of this study in Supplementary Table 4. In addition, XMLs, MCC and ML trees, and GISAID acknowledgement tables are also available in a GitHub repository (https://github.com/mostmarmot/Peru_AIV/).

Research involving human participants, their data, or biological material

Policy information about studies with human participants or human data. See also policy information about sex, gender (identity/presentation), and sexual orientation and race, ethnicity and racism.

Reporting on sex and gender	n/a
Reporting on race, ethnicity, or other socially relevant groupings	n/a
Population characteristics	n/a
Recruitment	n/a
Ethics oversight	n/a

Note that full information on the approval of the study protocol must also be provided in the manuscript.

Field-specific reporting

Please select the one below that is the best fit for your research. If you are not sure, read the appropriate sections before making your selection.

☒ Life sciences ☐ Behavioural & social sciences ☐ Ecological, evolutionary & environmental sciences

For a reference copy of the document with all sections, see nature.com/documents/nr-reporting-summary-flat.pdf

Life sciences study design

All studies must disclose on these points even when the disclosure is negative.

Sample size	Samples were collected in an outbreak situation where mass die-offs were taking place in sea birds and marine mammals. Sampling was conducted opportunistically by veterinarians working for the Forestry and Wildlife Service (SERFOR, which is the Peruvian government authority with jurisdiction over wildlife), in deceased animals that washed onto shores or in severely sick animals in the process of dying on shores.
Data exclusions	No data exclusions
Replication	We have collected multiple different types of samples from each individual animal sampled. All samples received until the time of this report have been tested in the lab, with varying degrees of success because different tissues had different viral loads. However, samples derived from single positive individuals were always positive, regardless of the tissue tested, and regardless of replicate (every samples tested at least twice). In other words, we were always able to replicate the results. NGS has been conducted on the sample with the highest viral load per individual. In a few cases, we've conducted NGS on multiple samples from a single individual animal. Only the best samples with the highest sequencing quality are reported in the manuscript and the genomic data generated has been deposited in public repositories like GenBank or GISAID.
Randomization	Randomization was not relevant to the study because all samples were collected "opportunistically" in the context of a disease outbreak of unknown etiology at the time. Vets from the Peruvian government and WCS-Peru collected as many samples as possible, from as many individuals as possible, during mass die-offs where hundreds of dead and diseased animals were washing onto shores throughout the coast of Peru. The lab tested all samples received.
Blinding	This is a single-blinded study. Sample collection was performed by vets from the Peruvian government and WCS-Peru, whereas sample processing was carried out by blinded laboratory personnel at PUCP. All samples were collected codified.

Reporting for specific materials, systems and methods

We require information from authors about some types of materials, experimental systems and methods used in many studies. Here, indicate whether each material, system or method listed is relevant to your study. If you are not sure if a list item applies to your research, read the appropriate section before selecting a response.

Materials & experimental systems

n/a	Involved in the study
☒	☐ Antibodies
☒	☐ Eukaryotic cell lines
☒	☐ Palaeontology and archaeology
☐	☒ Animals and other organisms
☒	☐ Clinical data
☒	☐ Dual use research of concern
☒	☐ Plants

Methods

n/a	Involved in the study
☒	☐ ChIP-seq
☒	☐ Flow cytometry
☒	☐ MRI-based neuroimaging

Animals and other research organisms

Policy information about studies involving animals; ARRIVE guidelines recommended for reporting animal research, and Sex and Gender in Research

Laboratory animals	This study did not involve laboratory animals.
Wild animals	Sampled animals (28) were opportunistically found during the avian influenza outbreak in Peru. Ages included adults, sub-adults, juveniles, pups, and a newborn (aborted dead) (detailed information provided in Supplementary Table 1). Sampled species were: Delphinus delphis, Pelecanus thagus, Calidris alba, Phalacrocorax bougainvillii, Larus becheri, Spheniscus humboldti and Otaria flavescens. All sampled animals were clinically sick (showing severe depression which allowed physical restraint) or recently deceased; thus, no wild animal was kept in captivity because of this study. All animals found alive were severely clinical ill and had to be humanely euthanized according to the AVMA guidelines for the euthanasia of animals: 2020 edition (Underwood & Anthony). Number of sampled animals were: Delphinus delphis (1), Pelecanus thagus (4), Calidris alba (1), Phalacrocorax bougainvillii (2), Spheniscus humboldti (1), Laurus becheri (1) and Otaria flavescens (18).
Reporting on sex	As sampling was opportunistically performed, it did not target any specific sex or sex ratio. Moreover, influenza virus was detected in both male and female animals. Association between sex and virus detection was not statistically assessed as this study was not designed for such purpose and samples were not balanced by sex.
Field-collected samples	Once collected, samples (swabs) were kept at 4-8°C within cooler boxes filled with frozen "ice packs" during 4 to 8 hours until arriving from field to lab. At the laboratory samples were either immediately processed or frozen at -80°C for long term storage.
Ethics oversight	Due to the fact that samples were collected as part of official duties during emergency response to avian influenza outbreak performed by Peruvian government authority´s veterinarians, no ethical approval was required for such activity. Nevertheless, we subsequently obtained approval anyway. All relevant permits and authorizations have been cited in the manuscript.

Note that full information on the approval of the study protocol must also be provided in the manuscript.
